# General-purpose and medically adapted large language models versus oncologist consensus for immunotherapy videos

**DOI:** 10.3389/fpubh.2026.1962857

**Published:** 2026-10-30

**Authors:** Şendağ Yaslikaya, Şuheda Ataş İpek, Esra Erdem Palaz

**Affiliations:** 1 Department of Medical Oncology, Giresun Training and Research Hospital, Giresun, Türkiye; 2 Department of Medical Oncology, Faculty of Medicine, Çukurova University, Adana, Türkiye; 3 Department of Medical Oncology, Bartın State Hospital, Ministry of Health, Bartın, Türkiye

**Keywords:** artificial intelligence, digital public health, health communication, health information quality, immune-related adverse events, immunotherapy, large language models, patient education

## Abstract

**Introduction:**

Patients receiving immune checkpoint inhibitors depend on accurate education to recognize immune-related adverse events and to know when to contact their oncology team, and many rely on online video of highly variable quality. Expert review of such material is resource-intensive, and large language models (LLMs) are increasingly proposed to support preliminary screening; whether any model agrees with specialist judgment closely enough to do so safely, and how often models pass expert-inadequate material as adequate, is unknown.

**Methods:**

In this cross-sectional content analysis (reported per STROBE), we evaluated anonymized transcripts of 90 English-language YouTube videos on cancer immunotherapy. Three medical oncologists and four LLMs — ChatGPT-5.5, Claude Opus 4.8, Gemini 3.1 Pro, and the medically adapted MedGemma 27B — independently scored every transcript using DISCERN, the Global Quality Score (GQS), and the Patient Education Materials Assessment Tool (PEMAT) understandability and actionability domains, each model in three separate runs, with expert majority consensus as the reference standard.

**Results:**

Agreement among oncologists was good to excellent, whereas agreement among models was limited. By expert-majority classification, 35 of 90 transcripts (38.9%) met at least three of the four adequacy criteria and 24 (26.7%) met all four. ChatGPT-5.5 deviated least from expert scores on every instrument and classified adequacy most accurately (sensitivity 94.3%, 95% CI 81.4–98.4; specificity 89.1%, 78.2–94.9; Cohen’s kappa 0.82, 0.69–0.93). Gemini and MedGemma scored systematically more leniently under the tested conditions and produced 43 and 26 false-positive adequacy classifications, respectively, passing material the oncologists judged inadequate; the medically adapted model did not improve agreement in this evaluation, and raw popularity metrics did not identify high-quality content.

**Discussion:**

The transcript-based educational content of most sampled videos did not meet all four adequacy criteria. These findings support further evaluation of selected model–prompt combinations for preliminary review of patient-education material, but they do not establish an operating threshold for clinical use, and the leniency observed in two models means expert review cannot be removed from the process.

## 1 Introduction

Immune checkpoint inhibitors have transformed the treatment of many malignancies, but their use can be complicated by immune-related adverse events affecting almost any organ system. Early recognition matters because symptoms are often mild or nonspecific at onset yet may progress to require treatment interruption or hospitalization (1). Patients are usually the first to notice these symptoms, outside scheduled visits. Education that tells patients which symptoms warrant a call to the oncology team is therefore part of safe treatment.

Patients and families increasingly obtain such education from online video. YouTube can explain complex topics effectively, but its content undergoes no standardized review and varies widely in accuracy, comprehensibility, and practical value (2–6). Poor scores are not the whole problem. Content that appears credible while omitting warning symptoms or instructions on when to seek help may create false reassurance. Established instruments — the 16-item DISCERN instrument (7), the Global Quality Score (GQS) (8), and the Patient Education Materials Assessment Tool (PEMAT) (9, 10) — capture complementary dimensions of reliability, educational value, understandability, and actionability.

Reviewing patient-facing material with expert raters is resource-intensive, and cancer-education programs curate more material than specialists can realistically assess. Large language models (LLMs) are increasingly proposed for this task (11, 12), and medically adapted models such as MedGemma are now available. How a model applies a structured instrument also depends on how it is prompted. Instruction-based, role-defining, domain-specific, rubric-anchored, few-shot, and step-by-step reasoning prompts can change the output of the same model (13, 14), evaluation prompts that walk the model through explicit criteria have improved agreement with human raters (15), and even formatting changes that leave the meaning of a prompt intact can alter performance (16). In a study that compared prompts for rating YouTube health videos with DISCERN, a prompt containing detailed scoring guidelines improved agreement with experts, although models still scored systematically higher than the experts (17). Early studies benchmarking a single general-purpose model against expert ratings of health-education video quality have reported only moderate agreement (18), and whether any model agrees with specialist judgment closely enough to support screening — and whether medically adapted models perform better — remains unclear. We therefore asked three medical oncologists and four LLMs to assess the same anonymized transcripts, quantified how far each model departed from expert consensus, and examined how often models classified expert-inadequate material as adequate. The comparison included a medically adapted model, extending its assessment to appraising the quality of patient-education material, a task distinct from the medical question-answering and image-interpretation tasks on which such models are usually evaluated.

## 2 Materials and methods

This cross-sectional content analysis is reported in accordance with the STROBE statement. Extended methods, including the full search strategy, eligibility criteria, anonymization rules, and additional model-access details, are provided in the Supplementary material.

### 2.1 Video selection and transcripts

On 29 May 2026, a single researcher searched YouTube from Türkiye in a browser that was not signed in to any Google or YouTube account and without a VPN, after clearing the browsing history. Ten prespecified queries were used: “what is immunotherapy,” “cancer immunotherapy,” “immunotherapy for cancer patients,” “immunotherapy side effects,” “immune checkpoint inhibitor side effects,” “immunotherapy adverse effects,” “cancer immunotherapy patient education,” “what to watch for during immunotherapy,” “immunotherapy treatment side effects,” and “immunotherapy patient information.” The first 50 standard results returned for each query were screened. Eligible videos were in English, were intended for patients with cancer or their caregivers, addressed the definition, indications, adverse effects, or warning symptoms of immunotherapy, and contained sufficient spoken material to generate a meaningful transcript. Videos unrelated to oncology, solely promotional material, duplicates, and videos without usable audio were excluded. After deduplication and eligibility screening, 90 videos were retained (Supplementary Figure S1). Screening of the search results was performed by one researcher; a second oncologist verified the eligibility of all included videos, but excluded results were not re-checked. Of the 500 results screened, 410 were excluded as duplicates or ineligible; reason-specific exclusion counts were not retained.

For each included video, the URL, duration, upload date, numbers of views, likes and comments, channel type, and description were recorded in a separate data form. Sources were classified as medical education platforms, public hospitals, public universities, private hospitals or universities, media channels, international medical associations, patient associations, or individual health-professional channels. These metadata were used only for descriptive and exploratory analyses and were not shown to any rater. Transcripts were obtained through YouTube’s transcript function and used verbatim without terminology correction or language editing. URLs, titles, channel names, speaker and institution names, popularity indicators, and other platform metadata were withheld; expressions within the transcript that could reveal the speaker or institution were masked without altering the medical content. Each transcript was assigned a study code (V001 onward). Raters therefore saw the spoken content and nothing else.

### 2.2 Raters and instruments

Three medical oncologists, each with at least 3 years of specialist experience, independently scored every transcript without access to one another’s ratings. One oncologist prepared the URL list and transcripts, and a second verified URL eligibility, transcript–video correspondence, and transcript preparation. The same anonymized transcripts, presented in the same order, were evaluated by three general-purpose large language models (ChatGPT-5.5, Claude Opus 4.8, and Gemini 3.1 Pro) and one medically adapted model (MedGemma 27B; Hugging Face checkpoint google/medgemma-27b-text-it). All model evaluations were performed on 29 May 2026. The general-purpose models were accessed through their public web interfaces with default settings; MedGemma was run in Google Colab through Hugging Face with the default generation settings of the checkpoint.

Every model received the same English-language prompt, reproduced in Supplementary Section S2. The prompt assigned the model the role of a medical oncology specialist; set out the criteria and scoring rules of each instrument and required the model to restate them before scoring; instructed it to rely solely on the text of the transcript, not to consult external internet sources, and not to supply information absent from the transcript from its own knowledge; required the scores to be clear, objective and justified; and specified the output as a summary table reporting, for each transcript, the DISCERN total, the GQS, and the PEMAT understandability and actionability percentages. Item-level scores were not requested, and only instrument totals were retained. The prompt set out the criteria and scoring rules of each instrument but did not reproduce the published item wording, which the expert raters had in front of them. In each run with the three web interfaces, the prompt was given at the start of a new session and the transcripts were then submitted sequentially, in the same order, within that session; each of these models completed three runs in separate sessions, with conversation memory cleared before each run. MedGemma was run in a notebook rather than a chat interface: a script implementing the same prompt was written in Google Colab, and the transcripts were then evaluated one at a time, each in a separate generation call consisting of the prompt followed by that transcript alone, with no conversation history carried from one transcript to the next; this procedure was repeated in each of the three runs. The three run scores were then averaged and rounded to the scoring granularity of the instrument — whole points for DISCERN and the GQS, whole percentage points for the PEMAT domains — giving one score per model and transcript. This is how the averaged scores were recorded in the study data form; it was not a prespecified analytical decision, and the mean absolute errors and the composite classification analyses were therefore repeated with the unrounded run means as a sensitivity analysis (Supplementary Table S7). MedGemma was run in Google Colab with the same prompt. Whether search and tool access were disabled was not verified for any web interface on the evaluation date, and default reasoning depth was not controlled; MedGemma had no retrieval or tool access. The prompt was piloted on the first 10 transcripts; no changes were required, the pilot outputs were not used in the analysis, and all 90 transcripts, including those 10, were rescored from the beginning in each of the three runs. Expert scores were not consulted during the pilot. Access conditions are given in Supplementary Table S1.

All raters applied the 16-item DISCERN instrument, each item scored from 1 (definitely no) to 5 (definitely yes), giving a total of 16–80 (7); the GQS, a single global rating from 1 to 5 (8); and the understandability and actionability domains of the PEMAT (9, 10). DISCERN assesses reliability, balance, uncertainty, treatment alternatives, and support for decision-making; the GQS provides an overall judgment of completeness, organization, clarity, and usefulness. PEMAT-Understandability assesses whether patients can process and explain the information, whereas PEMAT-Actionability assesses whether the material identifies feasible actions. No item was removed from any instrument in advance. Because the study was transcript based, all raters — the three oncologists and the four models — were instructed to base their assessment on the transcript text alone and to disregard visual and auditory material not reflected in it. The primary outcome was agreement between each model and expert consensus across the four instruments, with mean absolute error on DISCERN prespecified as the principal comparison. Secondary outcomes were inter-expert and inter-model agreement, adequacy classification, systematic bias, and associations between video characteristics and expert-rated quality.

### 2.3 Statistical analysis

Analyses used IBM SPSS Statistics 25.0 and R 4.4.1 with the irr, psych, irrCAC, blandr, and boot packages; the additional analyses performed during revision (run-level repeatability, PABAK, threshold sensitivity, and the interval estimates for the composite classification analyses) used Python 3 with NumPy and SciPy. Because neither library provides an intraclass correlation function, ICCs in those analyses were computed directly from the two-way mean-square decomposition of the rating matrix and were cross-checked against the irr package in R on the same data. Unless stated otherwise, confidence intervals were obtained by nonparametric bootstrap resampling of transcripts with 2,000 resamples and are reported as percentile intervals. Distributional assumptions were assessed using the Shapiro–Wilk test and graphical methods. Normally distributed variables are reported as mean ± standard deviation and non-normally distributed variables as median and interquartile range; categorical variables are reported as counts and percentages. The unit of analysis was the video (*n* = 90). The 2,520 analytical rating cells (90 videos × seven raters × four instruments) were derived after averaging 4,320 raw model-rating cells (90 videos × four models × three runs × four instruments); including the 1,080 expert-rating cells, the dataset comprised 5,400 raw rating cells overall. These were repeated, non-independent ratings and were not treated as independent observations. Expert consensus was the mean of the three oncologists for continuous analyses and their majority vote for binary classification.

The primary outcome was agreement between each model and expert consensus, quantified principally by mean absolute error (MAE), with DISCERN prespecified as the principal instrument. Agreement was characterized further by two-way intraclass correlation coefficients based on absolute agreement, reported as ICC(A,1) for every rater group and, for the three-oncologist reference panel, also as ICC(A,k), each with 95% bootstrap confidence intervals, and by Bland–Altman analysis with 95% limits of agreement (mean difference ± 1.96 SD) and bootstrap confidence intervals for the mean bias (Supplementary Table S2). A leave-one-expert-out analysis compared each oncologist with the mean of the other two, giving a between-expert error band against which model error was interpreted. This band is a descriptive benchmark derived from the same three-rater panel, not an external criterion of acceptable error. Model errors were compared by Friedman test with Kendall’s W, followed where significant by Wilcoxon signed-rank tests with Holm correction across the three comparisons with ChatGPT-5.5. Within-model repeatability across the three runs was quantified with ICC(A,1) and 95% bootstrap confidence intervals and with the within-transcript standard deviation. Because the averaged model score was rounded to the granularity of each instrument, the mean absolute errors and the composite classification analyses were repeated with the unrounded run means as a sensitivity analysis. Because model scores were averaged over three runs whereas each oncologist rated each transcript once, MAE and composite classification were also computed for each single run as a sensitivity analysis and compared with the between-expert MAE, which is based on single expert ratings.

Prespecified adequacy thresholds were DISCERN ≥ 51, GQS ≥ 4, and PEMAT ≥ 70%. All DISCERN analyses used the 16-item total score (range 16–80). The cut-off of 51 corresponds to the lower bound of the “good” band in a categorization widely applied to the DISCERN total score in studies of online video quality (very poor 16–26, poor 27–38, fair 39–50, good 51–62, excellent ≥ 63) (19, 20); GQS ≥ 4 denotes good-to-excellent overall quality (8); and PEMAT ≥ 70% is the threshold set by the PEMAT developers (9). These thresholds are operational conventions rather than validated clinical decision boundaries; the PEMAT developers noted that the 70% threshold lacks an empirical basis. The adequacy thresholds were applied by the investigators to the recorded scores after all scoring was complete and were not disclosed to the raters: no rater, expert or model, was told which score would count as adequate. The expert reference standard was formed instrument by instrument: a transcript was expert-adequate on an instrument when at least two of the three oncologists scored it at or above that instrument’s threshold, and the resulting four binary decisions were then counted. A transcript was leniently adequate when at least three of four thresholds were met and strictly adequate when all four were met. These composite definitions were investigator-defined and prespecified; no validated composite rule exists. Their robustness was examined in a sensitivity analysis spanning DISCERN 39–63, GQS 3–4, and PEMAT 60–80% under both definitions (180 combinations). Binary performance was summarized using sensitivity, specificity, Cohen’s kappa, prevalence- and bias-adjusted kappa (PABAK), and false-positive and false-negative counts. Because the reference standard was the majority judgment of three oncologists rather than an external gold standard, these quantities describe positive and negative agreement with expert consensus rather than diagnostic accuracy. Wilson intervals were used for sensitivity, specificity and PABAK, and 95% confidence intervals for Cohen’s kappa were estimated by bootstrap resampling of transcripts. A false positive was a model classifying as adequate a transcript that the expert majority classified as inadequate; this was interpreted as a potential false-reassurance signal in a screening context, not as evidence of clinical harm. Associations of popularity and duration with expert scores were assessed using Spearman correlation. All tests were two-sided, *p* < 0.05 indicated statistical significance, and exploratory association analyses were not adjusted for multiple comparisons. No video was excluded from analysis.

## 3 Results

The 90 videos had a median duration of 3.4 min (IQR 1.5–17.5), a median of 958.5 views (IQR 281.5–3,772.5), and a median of 8 likes (IQR 2–34.8). They were published between 2014 and 2025, with a median publication year of 2019. Patient associations and media channels were the most frequent source categories (28.9% each), followed by medical education platforms (14.4%) and public universities (13.3%). Overall, academic or professional sources accounted for 40.0% of the sample and consumer-oriented sources for 60.0%. Most videos (71.1%) addressed adverse-event recognition and awareness, 24.4% addressed adverse-event management, and 4.4% provided general or agent-specific overviews. Full descriptive characteristics of the sample are shown in Table 1. Seven raters generated 2,520 analytical rating cells after the three model runs were averaged; the underlying dataset comprised 4,320 raw model-rating cells and 5,400 raw rating cells overall.

**Table 1:** Descriptive characteristics of the included videos (*n* = 90).

Characteristic	Value
Number of videos, *n*	90
Duration, minutes, median (IQR)	3.4 (1.5–17.5)
Views, median (IQR)	958.5 (281.5–3,772.5)
Likes, median (IQR)	8.0 (2.0–34.8)
Publication year, median (range)	2019 (2014–2025)
Source: patient associations	26 (28.9%)
Source: media channels	26 (28.9%)
Source: medical education platforms	13 (14.4%)
Source: public universities	12 (13.3%)
Source: medical societies	5 (5.6%)
Source: public hospitals	3 (3.3%)
Source: private hospitals	3 (3.3%)
Source: individual channels	2 (2.2%)
Grouped source: academic/professional	36 (40.0%)
Grouped source: consumer-oriented	54 (60.0%)
Content: adverse-event recognition/awareness	64 (71.1%)
Content: adverse-event management	22 (24.4%)
Content: overview/agent-specific/other	4 (4.4%)

IQR, interquartile range. Academic/professional sources comprised medical education platforms, public and private universities and hospitals, and medical societies; consumer-oriented sources comprised media channels, patient associations, and individual channels.

### 3.1 Adequacy of the educational content

By expert-majority consensus, 45 transcripts (50.0%) met the DISCERN threshold, 37 (41.1%) the GQS threshold, 50 (55.6%) the PEMAT-Actionability threshold, and 32 (35.6%) the PEMAT-Understandability threshold. Thirty-five (38.9%) met at least three of four criteria and 24 (26.7%) met all four (Table 2). Understandability was the weakest dimension: a transcript could be reliable and still be hard to follow.

**Table 2:** Adequacy of video transcripts according to expert-majority consensus.

Criterion	Threshold	Adequate, *n*/90	%
DISCERN	≥ 51	45	50.0
GQS	≥ 4	37	41.1
PEMAT-Actionability	≥ 70	50	55.6
PEMAT-Understandability	≥ 70	32	35.6
Overall lenient	≥ 3/4 criteria	35	38.9
Overall strict	4/4 criteria	24	26.7

Expert consensus was the majority decision of the three medical oncologists. A video was rated adequate when it met the instrument-specific threshold shown; composite definitions required ≥ 3 of 4 criteria (lenient) or all 4 (strict). GQS, Global Quality Score; PEMAT, Patient Education Materials Assessment Tool.

### 3.2 Rater agreement

The models separated on the raw scores before any threshold was applied. Expert mean DISCERN scores ranged from 49.7 to 53.1; ChatGPT-5.5 (50.7 ± 11.9) and Claude (50.7 ± 11.2) closely matched that distribution, whereas MedGemma (62.5 ± 9.8) and Gemini (64.6 ± 10.3) assigned substantially higher scores. The same direction held for GQS and both PEMAT domains, so the leniency was not confined to one instrument or one cut-off. Full descriptive statistics by instrument are shown in Table 3, and the full score distributions by rater are shown in Supplementary Figure S2.

**Table 3:** Descriptive statistics of the raters by instrument (mean ± SD).

Instrument	Expert consensus	ChatGPT-5.5	Claude	Gemini	MedGemma
DISCERN	51.62 ± 13.00	50.72 ± 11.87	50.69 ± 11.17	64.64 ± 10.25	62.54 ± 9.83
GQS	3.17 ± 0.96	3.43 ± 0.84	3.32 ± 0.79	4.11 ± 0.69	3.64 ± 0.50
PEMAT-Actionability	72.66 ± 17.54	71.97 ± 16.80	69.54 ± 15.74	91.02 ± 7.16	80.06 ± 7.25
PEMAT-Understandability	54.52 ± 31.56	49.91 ± 34.04	48.74 ± 30.06	63.64 ± 21.87	61.94 ± 18.79

Expert consensus was the mean score of the three medical oncologists. SD, standard deviation; GQS, Global Quality Score; PEMAT, Patient Education Materials Assessment Tool.

Inter-expert reliability was good to excellent on the average-measure coefficients that correspond to the three-rater panel used as the reference standard, with ICC(A,k) of 0.89 (95% CI 0.84–0.93) for DISCERN, 0.91 (0.88–0.94) for the GQS, 0.95 (0.93–0.97) for PEMAT-Actionability, and 0.96 (0.93–0.97) for PEMAT-Understandability; the corresponding single-measure coefficients, which describe one oncologist rating alone, ranged from 0.74 to 0.88 (Figure 1). Agreement among the three general-purpose models was by contrast poor to moderate [ICC(A,1) 0.22–0.54], and adding MedGemma did not improve it (0.21–0.56) (Figure 1). These inter-model coefficients were computed on each model’s three-run mean, so they describe agreement between averaged scores rather than between individual runs. One model’s rating cannot stand in for another’s.

**Figure 1:**
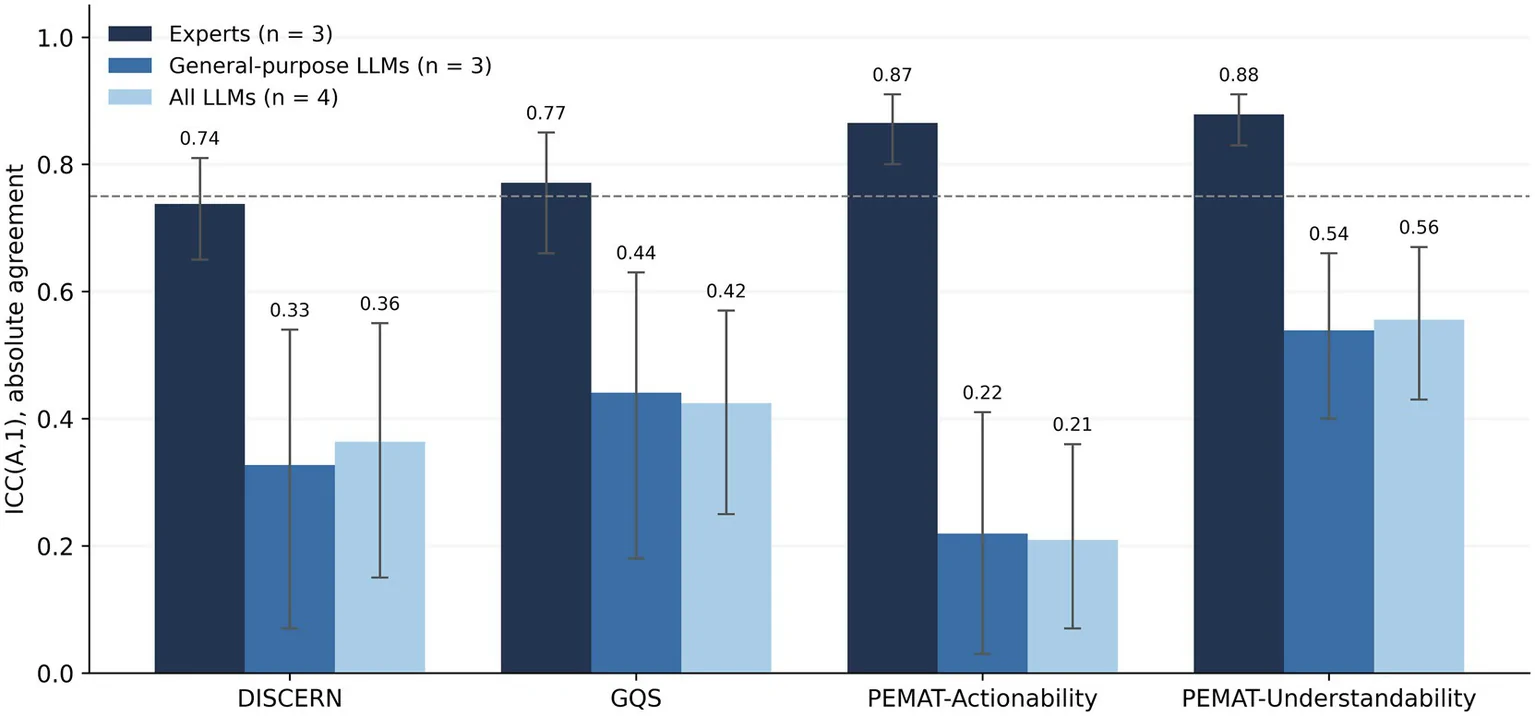
Single-measure absolute-agreement intraclass correlation coefficients [ICC(A,1)] among the three experts, among the three general-purpose LLMs, and among all four LLMs, by instrument. Error bars represent 95% bootstrap confidence intervals from 2,000 resamples of transcripts. The dashed horizontal line indicates the conventional reference value of ICC = 0.75, above which agreement is usually described as good. Coefficients for the models were computed on each model’s three-run mean.

### 3.3 Deviation from expert consensus

Friedman tests showed significant differences between models on all four instruments (DISCERN χ^2^ = 115.4, GQS χ^2^ = 63.4, PEMAT-Actionability χ^2^ = 54.0, PEMAT-Understandability χ^2^ = 28.7; df = 3, all *p* < 0.001; Supplementary Table S3), with Kendall’s W of 0.43 for DISCERN, 0.23 for GQS, 0.20 for PEMAT-Actionability, and 0.11 for PEMAT-Understandability; the effect was therefore substantial for DISCERN and small for PEMAT-Understandability. ChatGPT-5.5 had the lowest MAE on every instrument and Claude ranked second throughout (Figure 2; Supplementary Table S3). On DISCERN the errors were 4.65 for ChatGPT-5.5, 5.63 for Claude, 13.58 for MedGemma, and 16.11 for Gemini; ChatGPT-5.5’s error was below the between-expert error of 6.51, whereas Gemini’s and MedGemma’s were more than twice it. On the primary DISCERN outcome, ChatGPT-5.5’s error was lower than Claude’s (mean paired difference 0.98, 95% CI 0.22–1.89), Gemini’s (11.46, 9.36–13.62), and MedGemma’s (8.93, 7.06–10.82); all three comparisons remained significant after Holm correction. On the other three instruments the Holm-corrected comparisons gave the same pattern, with one exception: ChatGPT-5.5’s error was smaller than Gemini’s and MedGemma’s on the GQS and on both PEMAT domains, and smaller than Claude’s on both PEMAT domains, but the difference from Claude on the GQS was not significant.

**Figure 2:**
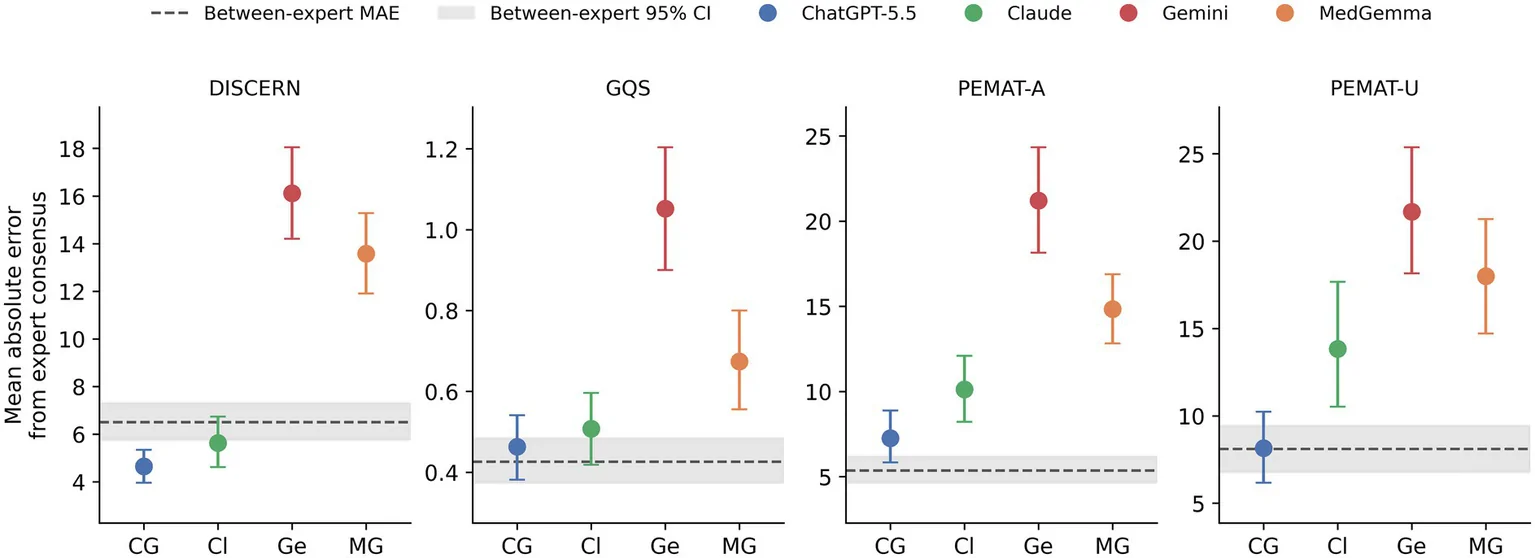
Mean absolute error of each model relative to expert consensus, by instrument. Points represent model-specific mean absolute errors, and error bars indicate 95% bootstrap confidence intervals. The dashed horizontal line and shaded band represent the between-expert mean absolute error and its 95% confidence interval, respectively; this band is a descriptive benchmark from the same three-rater panel, not an external criterion of acceptable error. Note that the panels use instrument-specific y-axis scales. Exact estimates and confidence intervals are provided in the Supplementary material. CG, ChatGPT-5.5; Cl, Claude Opus 4.8; Ge, Gemini 3.1 Pro; MG, MedGemma 27B.

Bland–Altman analysis showed that ChatGPT-5.5 and Claude had the smallest mean biases on DISCERN (−0.90 and −0.93). Gemini was positively biased on all four instruments (+13.03 on DISCERN, +0.94 on GQS, +18.36 on PEMAT-Actionability, +9.13 on PEMAT-Understandability), and MedGemma showed the same pattern less markedly (+10.93, +0.47, +7.40, +7.43). Bias estimates with 95% confidence intervals and limits of agreement are provided in Supplementary Table S2, and per-transcript Bland–Altman plots are shown in Supplementary Figure S3.

Run-to-run repeatability was high for DISCERN [ICC(A,1) 0.69–0.95] and both PEMAT domains (0.70–0.99) but lower for the GQS (0.38–0.72) (Supplementary Table S4). Single-run errors were modestly larger than those of averaged scores. On DISCERN, ChatGPT-5.5’s single-run MAE (mean 5.21, range 4.59–5.60) remained below the between-expert MAE (6.51), whereas on the GQS every model’s single-run MAE (0.68–1.13) exceeded the between-expert value (0.42). ChatGPT-5.5 had the lowest single-run MAE in 11 of 12 run–instrument combinations, and the lenient-composite ranking of models was unchanged in every run (Supplementary Tables S5a,b). Repeating every analysis with unrounded run means changed the estimates modestly and left the principal findings intact (Supplementary Table S7). Mean absolute errors moved by at most 0.14 units on any instrument, ChatGPT-5.5 remained the closest model on all four, and Gemini and MedGemma remained far outside the between-expert error. Under the lenient composite, Cohen’s kappa became 0.79 for ChatGPT-5.5, 0.57 for Claude, 0.41 for MedGemma and 0.13 for Gemini, with 6, 6, 25 and 42 false positives, preserving the ranking. Under the strict composite, ChatGPT-5.5 remained highest (0.86) but the order of the other three models changed (MedGemma 0.72, Gemini 0.67, Claude 0.66), so the strict-composite ordering below ChatGPT-5.5 is not robust to this choice.

### 3.4 Classification of adequacy and false reassurance

Under the lenient definition, ChatGPT-5.5 classified adequacy most accurately, with 94.3% sensitivity (95% CI 81.4–98.4), 89.1% specificity (78.2–94.9), Cohen’s kappa 0.82 (0.69–0.93), six false positives, and two false negatives. Claude showed 74.3% sensitivity (57.9–85.8) and 87.3% specificity (76.0–93.7) (kappa 0.62, 0.45–0.78). Gemini retained high sensitivity (91.4%, 77.6–97.0) but only 21.8% specificity (12.9–34.4), generating 43 false positives (kappa 0.11, −0.01 to 0.24); MedGemma showed 91.4% sensitivity (77.6–97.0), 52.7% specificity (39.8–65.3), and 26 false positives (kappa 0.39, 0.24–0.55) (Table 4; Figure 3A). Prevalence- and bias-adjusted kappa gave the same ranking (lenient definition: ChatGPT-5.5 0.82, Claude 0.64, MedGemma 0.36, Gemini −0.02; Table 4). Instrument-level sensitivity and specificity at each prespecified threshold were concordant (Supplementary Figure S4): on PEMAT-Actionability, Gemini’s specificity was 0% and MedGemma’s was 10%. Under the strict definition, ChatGPT-5.5 again had the highest kappa (0.86, 0.73–0.97).

**Table 4:** Composite adequacy classification performance against the expert-majority reference standard.

Definition	Model	Sensitivity (95% CI)	Specificity (95% CI)	Cohen’s κ (95% CI)	PABAK (95% CI)	FP	FN
Lenient (≥ 3/4)	ChatGPT-5.5	94.3 (81.4–98.4)	89.1 (78.2–94.9)	0.82 (0.69–0.93)	0.82 (0.67–0.91)	6	2
	Claude	74.3 (57.9–85.8)	87.3 (76.0–93.7)	0.62 (0.45–0.78)	0.64 (0.46–0.78)	7	9
	Gemini	91.4 (77.6–97.0)	21.8 (12.9–34.4)	0.11 (−0.01 to 0.24)	−0.02 (−0.22 to 0.18)	43	3
	MedGemma	91.4 (77.6–97.0)	52.7 (39.8–65.3)	0.39 (0.24–0.55)	0.36 (0.15–0.53)	26	3
Strict (4/4)	ChatGPT-5.5	91.7 (74.2–97.7)	95.5 (87.5–98.4)	0.86 (0.73–0.97)	0.89 (0.75–0.95)	3	2
	Claude	70.8 (50.8–85.1)	97.0 (89.6–99.2)	0.73 (0.53–0.88)	0.80 (0.64–0.89)	2	7
	Gemini	91.7 (74.2–97.7)	78.8 (67.5–86.9)	0.61 (0.43–0.77)	0.64 (0.46–0.78)	14	2
	MedGemma	87.5 (69.0–95.7)	87.9 (77.9–93.7)	0.71 (0.54–0.86)	0.76 (0.59–0.86)	8	3

Values are percentages unless otherwise stated. The lenient composite required ≥ 3 of 4 criteria (expert-adequate 35/90); the strict composite required all 4 (expert-adequate 24/90). Sensitivity, specificity and PABAK intervals are Wilson intervals; kappa intervals are percentile bootstrap intervals from 2,000 resamples of transcripts. Because the reference standard is an expert majority vote rather than an external gold standard, sensitivity and specificity describe positive and negative agreement with expert consensus. FP, false positive; FN, false negative; κ, Cohen's kappa; PABAK, prevalence- and bias-adjusted kappa (2 × observed agreement − 1).

**Figure 3:**
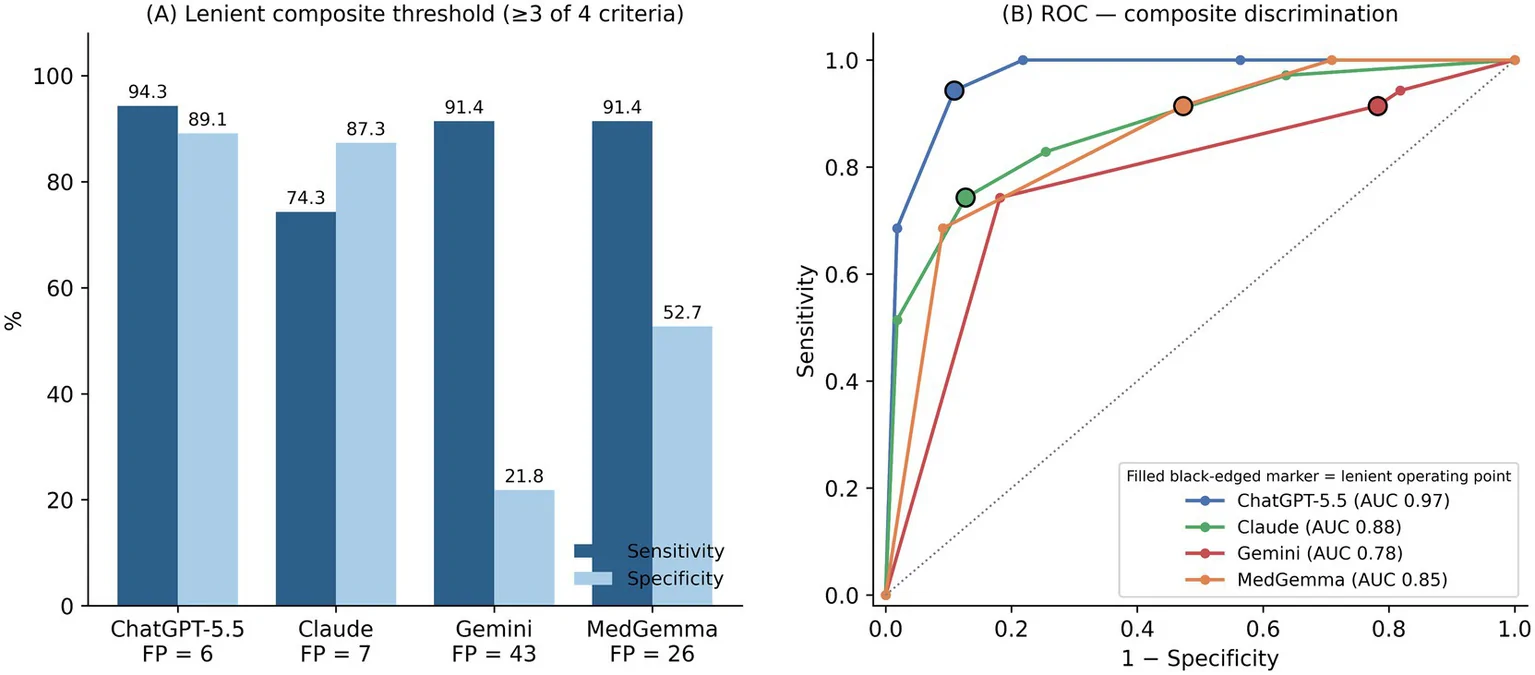
Composite adequacy classification against the expert-majority reference standard. **(A)** Sensitivity and specificity at the lenient composite threshold, defined as meeting at least three of four adequacy criteria; false-positive counts are shown for each model. **(B)** Receiver-operating-characteristic curves using the pooled number of met criteria (0–4) as the ordinal classification score against the lenient expert-majority reference standard. Areas under the curve were 0.97 for ChatGPT-5.5, 0.88 for Claude Opus 4.8, 0.78 for Gemini 3.1 Pro, and 0.85 for MedGemma 27B. The index score and the reference standard are derived from the same four thresholds, so these curves re-express the composite classification of Table 4 rather than providing independent validation; with only five possible score levels, the areas should be interpreted cautiously.

Areas under the receiver-operating-characteristic curve, computed on the pooled composite score (number of instrument thresholds met, 0–4) against the lenient expert-majority reference, were 0.97 for ChatGPT-5.5, 0.88 for Claude, 0.85 for MedGemma, and 0.78 for Gemini (Figure 3B). Because the index score and the reference standard are derived from the same four thresholds, this curve re-expresses the composite classification in Table 4 across its five possible operating points rather than providing independent validation, and the estimates should be interpreted accordingly. In the threshold sensitivity analysis (180 combinations), ChatGPT-5.5 had the highest kappa in 178 combinations (the two exceptions, both under the strict definition, favoured MedGemma), Gemini or MedGemma produced the most false positives in all 180, and ChatGPT-5.5 produced fewer false positives than both in all 180 (Supplementary Table S6). This is a sensitivity analysis within the same sample: it tests the stability of the relative ranking of the models, not the validity of the thresholds, and absolute agreement varied considerably across the scenarios reported in Supplementary Table S6 (ChatGPT-5.5 kappa 0.50–0.92). These counts describe the present dataset and are not inferential.

### 3.5 Exploratory findings

Raw popularity was unrelated to expert-assessed quality: expert DISCERN correlated neither with views (ρ = 0.07, *p* = 0.51) nor likes (ρ = 0.18, *p* = 0.09), and views were unrelated to GQS (ρ = 0.08, *p* = 0.43). Because raw counts are influenced by time online (median video age 2,392 days; range 175–4,398), age-adjusted measures were also examined. Views per day remained unrelated to DISCERN (ρ = 0.16, *p* = 0.13), whereas the likes-to-views ratio correlated modestly with DISCERN (ρ = 0.28, *p* = 0.009) and GQS (ρ = 0.29, *p* = 0.006). Duration correlated moderately with DISCERN (ρ = 0.59, *p* < 0.001), potentially because longer videos had more opportunity to address individual scale items. No general-purpose model showed strong exploratory agreement with MedGemma; ICC values across instruments ranged from 0.32 to 0.37.

## 4 Discussion

Only about a quarter of patient-facing immunotherapy videos met all four adequacy criteria, and understandability was the weakest domain. A video can be accurate and still never tell a patient which symptoms warrant a telephone call — the judgment that decides whether an immune-related adverse event is reported early (21). Since 60% of the sample came from consumer-oriented channels, and since the educational needs surrounding these events are already recognized as incompletely met (22), academic and professional organizations should be producing and curating more of this material themselves.

As screening aids the four models were not close to equivalent. ChatGPT-5.5 tracked expert consensus closely on every instrument and classified adequacy with substantial agreement, consistent with reports that AI can approximate human ratings of educational videos (18, 23, 24). Gemini and MedGemma, by contrast, were systematically lenient. Gemini passed 43 transcripts and MedGemma 26 that the oncologists judged inadequate. That is the number with practical consequences: in a curation workflow it would be the more damaging error, because deficient material would clear preliminary screening rather than adequate material merely being held back. This pattern matches the scoring bias described in the LLM-as-a-judge literature (12), and large-scale work cautions that reliability is not equivalent to validity (25).

The gap was large enough to matter for screening, and it is clearest on DISCERN and the GQS. Across the whole sample Gemini scored 13.03 DISCERN points and 0.94 GQS points above expert consensus and MedGemma 10.93 and 0.47 points above it, whereas ChatGPT-5.5 and Claude were within one point of the experts on DISCERN. Among the 55 transcripts the expert majority judged inadequate, the mean expert DISCERN score was 43.4 against 62.1 for Gemini and 59.0 for MedGemma, with ChatGPT-5.5 close to the experts at 44.0; because these transcripts were selected on low expert scores, the whole-sample bias is the more conservative estimate. PEMAT-Actionability showed the same direction (expert 63.6; Gemini 90.6, MedGemma 77.4, ChatGPT-5.5 65.4), although PEMAT percentages depend on how many items each rater treated as applicable, which was not recorded. The risk is systematic upgrading of deficient material, not noise. Correlation alone hides this; absolute error, bias and false-positive counts do not.

The medically adapted model showed no advantage under the conditions tested. This is a single comparison of one checkpoint under one prompt and does not identify a cause. MedGemma did not agree with expert consensus more closely than ChatGPT-5.5 or Claude; its errors were smaller than Gemini’s on several instruments, but medical adaptation conferred no consistent advantage, although domain fine-tuning improves factual medical question-answering (26, 27). Rating educational quality is a different task, requiring calibration across reliability, balance, understandability, and actionability rather than factual recall; benchmarking of clinical LLM agents points the same way (28). Previous oncology applications of MedGemma have concentrated on imaging and clinical text extraction (26, 29). A medical label is not evidence of safer evaluation.

Agreement between the models was poor to moderate [ICC(A,1) 0.21–0.56 across all four models]. One plausible contributor is that the expert raters scored from the published instrument forms, whereas the models were given summary criteria and scoring rules rather than the published item wording, and no item list at all for the PEMAT domains, and therefore applied each instrument as they represented it internally; differences between those internal representations would produce the pattern observed, in which the models agreed poorly with one another while one model tracked expert consensus closely. Because a rating from one model cannot be generalized to another, any program adopting such a tool must validate the specific model against local expert judgment, document the version used, and revalidate after updates (25).

These results should be read as properties of each model under the prompt used here, not as fixed limits of the models. The prompt assigned the model a specialist role, supplied the scoring rules of each instrument, required the scores to be clear, objective and justified, and specified a summary table of instrument totals as the output. It did not provide worked examples, require an item-by-item score to be reported, or require the transcript passage supporting each point to be quoted. More constrained prompting might reduce leniency. Requiring an item-level score with the supporting transcript passage for every point, stating explicitly that information not present must receive the lowest score, supplying the full published item wording, and adding a small number of expert-scored example transcripts are all plausible ways to lower false-positive adequacy classifications, and guided-scoring prompts have already improved DISCERN agreement in a related setting (17). Whether they would do so for Gemini and MedGemma was not tested here. The benefit of prompt changes is also model-specific and can be unstable (14), and a prompt tuned on expert-scored material must be validated on separate transcripts to avoid overstating agreement. Even an optimized prompt would therefore support, not replace, expert review: the appropriate role remains ranking or flagging material for human attention, with the specific model–prompt combination validated locally and revalidated after changes to either.

### 4.1 Strengths and implications for cancer education practice

Three design features support the internal comparison. The study compared three general-purpose models and a medically adapted model with three independent oncologists on identical anonymized inputs and a common prompt; it removed non-content cues by withholding channel prestige, speaker identity, popularity, and production quality; and it used four complementary instruments covering reliability, global educational value, understandability, and actionability. Repeated model scoring and a multidimensional agreement framework further reduced dependence on a single stochastic output or metric. These features strengthen the internal comparison between raters; they do not establish external validity for other languages, platforms, model versions, or clinical settings.

The role this supports is narrow. If prospectively validated, an LLM could be used by a cancer-education program to rank or flag material for human attention, reducing the volume that specialists must review, rather than to certify material as suitable. Because the costly error is the false positive, a model selected for this purpose should be chosen on specificity rather than on overall accuracy or sensitivity, and a deliberately conservative threshold is preferable to a balanced one. These data identify model–prompt combinations worth evaluating further; they do not establish an operating threshold for clinical use. Any such workflow should be preceded by local validation against a sample scored by the program’s own educators, since the agreement we observed for one model at one point in time cannot be assumed to hold for another model or after a version update. Understandability, the dimension on which the videos performed worst and on which models diverged most, warrants particular attention when material is selected for patients beginning immunotherapy.

### 4.2 Limitations

This study has substantial limitations. First, it included only English-language videos from one platform, identified on a single search date; YouTube rankings and available content change over time. Screening was performed by one researcher, and reason-specific exclusion counts were not retained, creating potential selection bias and preventing a fully enumerated screening flow. YouTube search results also depend on location and personalization; although the search was performed without account sign-in, results obtained from Türkiye may differ from those obtained elsewhere.

Second, the transcript-based design excluded graphics, animation, visual narration, audio quality, subtitles, and other audiovisual features. PEMAT results therefore represent modified transcript-based understandability and actionability, not the complete patient experience. The instrument version applied was not documented at the time of scoring, and because only domain percentages were retained the item set cannot be reconstructed from the data; the 70% adequacy threshold is therefore used as an operational convention rather than as the developers’ cut-off applied to the validated instrument, and the classification is reported at PEMAT thresholds of 60 and 80% alongside 70% (Supplementary Table S6), although varying the threshold does not itself quantify the effect of an unrecorded item set. Automatic YouTube transcripts were used verbatim apart from identity masking and may contain uncorrected transcription errors. Item applicability was left to each rater’s judgment: all raters were instructed to disregard visual and auditory material absent from the transcript, but whether a dependent item was recorded as not applicable or scored as not met was decided rater by rater, and only instrument totals were retained. Because PEMAT domain scores are percentages of applicable items, a rater who excluded more items was scored on a smaller denominator, so unrecorded differences in these decisions could have affected PEMAT percentages in either direction. We cannot separate how much of the difference between raters on PEMAT reflects their judgment of the content and how much reflects different item sets or denominators, and because two of the four adequacy criteria are PEMAT domains, this uncertainty carries into the composite classification as well.

Third, the three oncologists formed a small expert reference panel. Their consensus represents agreement with experienced clinicians rather than an external gold standard and should not be interpreted as absolute accuracy; because the reference standard is itself measured with error, the agreement statistics in Table 4 are best read as positive and negative agreement with expert consensus.

Fourth, model results are a time-specific snapshot. Public web interfaces do not expose decoding parameters, and MedGemma was run with the default generation settings of its checkpoint. Repeatability across runs was high for DISCERN and PEMAT but lower for the GQS, where single-run agreement with experts was weaker than averaged agreement; conclusions about GQS-level agreement therefore depend on averaging several runs. In the three web interfaces, transcripts within a run were scored sequentially in a single session, so earlier transcripts may have influenced the scoring of later ones, and the influence of the initial instructions may have weakened over a long session; the transcript order was identical across runs, so any order effect would be common to these three models rather than specific to one, although this does not guarantee that it acted equally on each. MedGemma, in which each transcript was scored in a separate call, is not subject to this mechanism; the session structure therefore differed between MedGemma and the three web interfaces, and that difference is confounded with model identity in the comparison. Because transcripts within a run were not independent, confidence intervals obtained by resampling transcripts may be narrower than the true uncertainty.

Fifth, a single prompt was used for all models; because LLM output is sensitive to prompt design, the results apply to the model, prompt and session conditions tested, and alternative prompting strategies might have produced different levels of agreement. The prompt described the criteria and scoring rules of each instrument but did not reproduce the published item wording, which the expert raters had in front of them; supplying the full item text might change both the agreement with experts and the agreement between models. Search and tool access in the consumer interfaces were not verified as disabled, and web retrieval could have allowed a model to identify the source video and so undermine blinding; anonymization reduced this risk, and the leniency pattern was also observed in MedGemma, which had no retrieval capability, but de-anonymization cannot be excluded. Prior exposure to the publicly available videos or related text during model development is also possible; such familiarity could encourage completion of omitted information and contribute to score inflation, but this mechanism could not be tested.

Finally, the adequacy thresholds are operational conventions, and the composite definitions have not been externally validated; the stability of the model ranking across the thresholds examined does not validate them. Readability was not measured, exploratory associations were not multiplicity-adjusted, and classification behavior rather than patient outcomes was evaluated. False positives therefore identify a plausible false-reassurance pathway, not demonstrated clinical harm.

## 5 Conclusion

The transcript-based educational content of most sampled cancer-immunotherapy videos did not simultaneously meet criteria for reliability, overall quality, understandability, and actionability. ChatGPT-5.5 showed the closest and most balanced agreement with oncologist consensus in this evaluation, although on the GQS that agreement depended on averaging three runs, since every model’s single-run GQS error exceeded the between-expert error. Gemini and MedGemma produced substantial score inflation and excess false-positive classifications. Certain models may warrant prospective evaluation as preliminary prioritization tools, but expert review must remain the final step, and any model adopted should be re-evaluated after version changes. Future studies should incorporate multilingual samples, direct audiovisual assessment, external expert panels, prospective evaluation across model updates, and patient-centered outcomes.

## Data Availability

The de-identified score matrix, the raw per-run model outputs from all three runs, video identifiers and URLs, the scoring prompt, the full 180-combination threshold sensitivity grid, and the analysis code are available from the corresponding author on reasonable request. Full transcripts are not redistributed owing to copyright restrictions. Further inquiries can be directed to the corresponding author.
