# Supplementary material for: General-purpose and medically adapted large language models versus oncologist consensus for immunotherapy videos
Source: Front Public Health. 2026 Oct 30;14:1962857. doi: 10.3389/fpubh.2026.1962857 (PMC13759737; doi:10.3389/fpubh.2026.1962857)
Supplement: Supplementary file 1 [file Supplementary_file_1.docx]

Manuscript ID 1962857 — "General-Purpose and Medically Adapted Large Language Models Versus Oncologist Consensus for Immunotherapy Videos"

**Supplementary Material**

# **Section S1. Supplementary Methods**

## **S1.1 Search strategy**

A search was conducted on YouTube on 29 May 2026 by a single researcher after clearing browsing history. The search was performed from Türkiye in a browser that was not signed in to any Google or YouTube account and without a VPN. Ten prespecified queries were used: "what is immunotherapy," "cancer immunotherapy," "immunotherapy for cancer patients," "immunotherapy side effects," "immune checkpoint inhibitor side effects," "immunotherapy adverse effects," "cancer immunotherapy patient education," "what to watch for during immunotherapy," "immunotherapy treatment side effects," and "immunotherapy patient information." For each query the first 50 videos returned in YouTube's standard ranking were screened (500 search-result positions before cross-query deduplication).

## **S1.2 Eligibility criteria**

Inclusion criteria: the video was intended to inform patients about immunotherapy; the content related to oncological disease; the video addressed at least one of the definition of immunotherapy, its indications, its side effects, or points requiring attention during treatment; the video was in English; and it contained sufficient spoken content to allow a transcript to be created.

Exclusion criteria: immunotherapy content unrelated to oncology; videos intended solely for advertising or promotion; duplicate videos; and videos with insufficient audio content or from which a meaningful transcript could not be generated. After deduplication and eligibility screening, 90 videos were retained. Screening of the search results was performed by one researcher; a second oncologist verified the eligibility of all included videos, but excluded results were not re-checked, and reason-specific exclusion counts were not retained.

## **S1.3 Metadata and source classification**

For each included video the URL, duration, upload date, view count, like count, comment count, channel type and description were recorded on a separate data form. This metadata was not provided to expert evaluators or to the models. Source channels were classified as medical education platforms, public hospitals, public universities, private hospitals or universities, media channels, international medical associations, patient associations, or individual channels run by physicians or other health professionals. Classification was performed separately from content scoring and outside the blinding process so that it could not influence transcript scores.

## **S1.4 Transcript preparation and anonymization**

Transcripts were obtained directly from YouTube's transcript feature. Verbatim spoken content was used without terminology standardization or linguistic editing. The only modification was the masking of expressions that could directly reveal the identity of the speaker or institution, performed in a way that did not compromise medical content. Transcripts excluded the video title, channel name, speaker identity, institution name, view count, like count, comments and all other platform metadata. All transcripts were labelled with unique codes (V001, V002, V003 and so on). Because the design was transcript based, all raters — the three oncologists and the four models — were instructed to base their assessment on the transcript text alone and to disregard visual and auditory material not reflected in it.

## **S1.5 Expert raters**

The three expert evaluators each had at least three years of experience in medical oncology. One expert prepared the URL list and transcripts; a second checked the selected URLs, transcript–video correspondence and transcript preparation. All three assessed the transcripts independently, without watching the videos and without access to one another's scores. Each expert scored every transcript once using all four instruments, working from the published instrument forms.

## **S1.6 Model access, versions and evaluation conditions**

All model evaluations were performed on 29 May 2026. The three general-purpose models were accessed through their public web interfaces, in which decoding parameters are not user-configurable and were therefore not recorded. MedGemma 27B (instruction-tuned) was run in Google Colab via Hugging Face with the default generation settings of the checkpoint. In each run with the three web interfaces, the prompt was entered at the start of a new session and the 90 anonymized transcripts were then submitted one after another, in the same order, within that session; each of these models completed three such runs in three separate sessions, with conversation memory cleared before each run. MedGemma, run in a notebook rather than a chat interface, was handled differently: a script implementing the same prompt was written in Google Colab, and the transcripts were then evaluated one at a time, each in a separate generation call consisting of the prompt followed by that transcript alone, with no conversation history carried between transcripts; this was repeated in each of the three runs. For each instrument, the three run scores were averaged and rounded to the scoring granularity of the instrument (whole points for DISCERN and the GQS, whole percentage points for the PEMAT domains) for the primary analyses; analyses with unrounded means are reported as a sensitivity analysis. Whether search and tool access were disabled was not verified for any web interface on the evaluation date, and default reasoning depth was not controlled; MedGemma had no retrieval or tool access. Full access conditions are given in Supplementary Table S1.

## **S1.7 Assessment instruments**

DISCERN comprises 16 items rated on a 5-point scale (possible range 16–80), with higher scores indicating more reliable, balanced and higher-quality health information. The Global Quality Score was rated from 1 to 5. PEMAT understandability reflected the extent to which patients could comprehend the information, and PEMAT actionability the extent to which the information could help patients take a concrete action. No item was removed from any instrument in advance. Where an item depended on visual or auditory material not reflected in the transcript, each rater decided whether to record it as not applicable or to score it as not met; this judgement was left to the individual rater, and because only instrument totals were retained, item-level applicability decisions cannot be recovered for any rater. Two points follow. First, PEMAT results are interpreted as modified transcript-based measures rather than as measures of full audiovisual quality, and the developers' 70% threshold is used as an operational convention rather than as that threshold applied to the validated instrument. Second, PEMAT domain scores are percentages of the applicable items, so a rater who treated more items as not applicable was scored on a smaller denominator.

## **S1.8 Outcomes**

The primary outcome was agreement between expert-consensus scores and the transcript-based scores generated by the three general-purpose models and MedGemma, characterized with complementary metrics — mean absolute error, intraclass correlation, Bland–Altman bias and adequacy classification — assessed separately for DISCERN, GQS, PEMAT-Understandability and PEMAT-Actionability. Secondary outcomes were inter-expert and inter-model agreement, adequacy-classification performance, and associations between video characteristics and expert-rated quality.

## **S1.9 Statistical detail**

Analyses used IBM SPSS Statistics 25.0 and R 4.4.1 with the irr, psych, irrCAC, blandr and boot packages; the additional analyses performed during revision (run-level repeatability, PABAK, threshold sensitivity, and the interval estimates in Table 4) used Python 3 with NumPy and SciPy. Because neither library provides an intraclass correlation function, ICCs in those analyses were computed directly from the two-way mean-square decomposition of the rating matrix and were cross-checked against the irr package in R on the same data. Because transcripts within a run were scored sequentially and are therefore not independent, confidence intervals obtained by resampling transcripts may be narrower than the true uncertainty. Unless stated otherwise, confidence intervals were obtained by nonparametric bootstrap resampling of transcripts with 2,000 resamples and are reported as percentile intervals.

Distribution of continuous variables was assessed with the Shapiro–Wilk test and graphical methods. Normally distributed variables are presented as mean ± standard deviation and non-normally distributed variables as median and interquartile range; categorical variables are reported as frequencies and percentages. The unit of analysis was the video (n = 90). The 2,520 analytical rating cells (90 videos × 7 raters × 4 instruments) were obtained after averaging the three repeated runs for each model; the dataset comprised 4,320 raw model-rating cells and 1,080 expert-rating cells (5,400 raw rating cells in total). These are repeated, non-independent ratings and were not treated as independent observations.

Absolute agreement among experts and among models was evaluated using a two-way intraclass correlation coefficient based on absolute agreement, with ICC(A,1) for single-rater agreement, reported for every rater group, and ICC(A,k) for average-rater agreement, reported for the three-oncologist reference panel, each with 95% bootstrap confidence intervals. Inter-model coefficients were computed on each model's three-run mean, so they describe agreement between averaged scores rather than between individual runs. Bland–Altman analysis reported mean bias with its 95% confidence interval and the 95% limits of agreement (Supplementary Table S2); proportional-bias slopes are not reported, because regressing the difference between two measurements on their mean yields a non-zero slope whenever the measurements differ in precision, which cannot be excluded here. A leave-one-expert-out analysis compared each expert with the mean of the other two, yielding a between-expert error band against which model error was interpreted; this band is a descriptive benchmark derived from the same three-rater panel, not an external criterion of acceptable error.

Model errors were compared using the Friedman test with Kendall's W; where significant, pairwise comparisons used the Wilcoxon signed-rank test with Holm correction across the three comparisons with ChatGPT-5.5. Within-model repeatability across the three runs was quantified with ICC(A,1) and with the within-transcript standard deviation (Supplementary Table S4), and MAE and composite classification were also computed for each single run (Supplementary Tables S5a and S5b).

For binary adequacy analyses, sensitivity, specificity, Cohen's κ, prevalence- and bias-adjusted κ (PABAK), and false-positive and false-negative counts were calculated. Because the reference standard was the majority judgement of three oncologists rather than an external gold standard, these quantities describe positive and negative agreement with expert consensus rather than diagnostic accuracy. Instrument-specific thresholds (DISCERN ≥ 51, GQS ≥ 4, PEMAT ≥ 70%) were prespecified. The adequacy thresholds were applied by the investigators to the recorded scores after all scoring was complete and were not disclosed to the raters: no rater, expert or model, was told which score would count as adequate. All DISCERN analyses used the 16-item total score; the cut-off of 51 corresponds to the lower bound of the "good" band in a categorization widely applied to the DISCERN total score in studies of online video quality (very poor 16–26, poor 27–38, fair 39–50, good 51–62, excellent ≥ 63). Ninety-five percent confidence intervals for sensitivity, specificity and PABAK used the Wilson method; those for Cohen's κ were estimated by bootstrap resampling of transcripts. The robustness of the adequacy thresholds and of the composite definitions was examined in a sensitivity analysis spanning DISCERN 39–63, GQS 3–4 and PEMAT 60–80% under both definitions (180 combinations; Supplementary Table S6).

# **Section S2. Scoring prompt, output, session procedure and pilot**

## **S2.1 The prompt used in this study**

The prompt reproduced in Section S2.2 is the prompt used in this study, taken from the authors' study records. It was prepared before scoring began and was entered unchanged at the start of each session. The same prompt was used for all four models and all three runs.

The prompt covers the following: the instruments applied; the assignment of a specialist role to the model; the requirement that the model restate the scoring criteria before evaluating; the instruction to rely on the transcript text alone, not to consult external internet sources, and not to supply information absent from the transcript; the instruction to treat items depending on material absent from the transcript as not applicable or as not met; the requirement that scores be clear, objective and justified; and the summary-table output format. The prompt set out the criteria and scoring rules of each instrument; it did not reproduce the published item wording of the instruments, which the expert raters had in front of them.

The raw per-run model outputs, from which all reported instrument totals were transcribed, were retained and are available from the corresponding author on reasonable request.

## **S2.2 Prompt (original language English)**

## As a medical oncology specialist, I want you to evaluate video transcripts that I will share with you using the DISCERN, GQS (Global Quality Score), and PEMAT (understandability and actionability) scales. While performing this analysis, you must strictly refrain from using any external internet sources, you must not complete missing information with your own external knowledge base, and you must not fill in parts not present in the transcripts on your own. You must rely solely on the current text provided within the transcript and evaluate only to the extent permitted by the available data.

## Important Constraint (Visual Elements): You will perform the evaluation solely based on the text content of the transcript. You will not include visual elements, graphics, animations, tables, or slides that may exist in the video but are not reflected in the transcript text. For elements not explicitly mentioned in the text, you will approach the relevant scale items directly as "Not Applicable / N/A" or "No (0)".

## Before moving on to the evaluation process, you will explain all the criteria and scoring rules you will use step-by-step in detail as follows:

## 1. DISCERN Scoring and Criteria. Criteria Description: Consists of 16 core questions evaluating the quality of written or verbal information regarding health and treatment choices, publication reliability, and whether alternatives are presented neutrally and in a balanced manner. Scoring System: Each item is scored from 1 (definitely no — criterion not met at all) to 5 (definitely yes — criterion completely met).

## DISCERN Criteria — Section 1: Reliability. This section assesses whether the source of the information is trustworthy and whether it is presented impartially. (1) Clear aims: are the aims of the publication or content clearly stated? (2) Relevance to the user: is the information suited to the needs of the people seeking or using it (patients or their relatives)? (3) Sources stated: is the source of the information (author, institution, and so on) clearly stated? (4) Date of the information: is it clear when the information was produced or last updated? (5) Balance and impartiality: is the information presented in a balanced and unbiased way? (6) Additional sources of information: are additional sources from which the user could obtain further information indicated? (7) Handling of uncertainty: are areas of uncertainty in the treatment field (for example side effects and unknowns) addressed honestly?

## DISCERN Criteria — Section 2: Specific details about treatment choices. This section examines how clearly the treatment alternatives and their consequences are explained. (8) Existence of options: are different treatment options mentioned? (9) The option of no treatment: are the consequences of receiving no treatment or of watchful waiting addressed? (10) Quality of each option: is it described in detail how each option works and what its benefits are? (11) Risks and side effects: are the risks and possible side effects of the treatments clearly stated? (12) Effect on quality of life: are the effects of the chosen treatments on the patient's daily life and quality of life considered? (13) Comparison of alternatives: are the different treatment options compared with one another? (14) Shared decision-making: is the patient encouraged and supported in bringing their own preferences and values into the treatment process?

## DISCERN Criteria — Section 3: Overall quality of the publication. (15) Support for a shared decision: is the information structured so as to support the patient in reaching an informed decision together with their doctor? (16) Overall assessment of quality: taking all of these criteria into account, how is the information quality of the source assessed as a whole? (This final item is the overall judgement that is reflected in the instrument's total score.)

## 2. GQS (Global Quality Score) Scoring and Criteria. Criteria Description: A single-item rating scale that evaluates the overall quality of the source for patients, its usefulness, flow, organization, and informative value in a holistic manner. Scoring System: Scored as a single integer from 1 to 5. 1 = Poor quality: content with serious deficiencies, almost unusable or of no use to patients. 2 = Fair-to-poor quality: some useful parts, but with important gaps and inadequacies. 3 = Moderate quality: an average level of information, with some useful parts but also clear shortcomings. 4 = Good quality: generally well structured, and quite useful and informative for patients. 5 = Excellent quality: ideal for patients, flawless, highly informative and prepared to a professional standard.

## 3. 3. PEMAT Scoring and Criteria (Understandability and Actionability). PEMAT is a standard tool developed to assess two critical dimensions of written or audiovisual patient education material: understandability and actionability.

## Understandability (PEMAT-U): Measures how easily the target audience (patients) can comprehend the content in terms of language, word choice, organization, and flow. The main elements examined are: Content and structure — the main message is clear and the content is organized under logical headings and subheadings; Language and expression — medical jargon is avoided, or technical terms that are used are explained in plain language, and a style close to everyday speech is used; Layout and presentation — the text is arranged so as to be easy to read (page layout, use of lists, and so on); Summarizing — complex sections or key points are summarized and reinforced.

## Actionability (PEMAT-A): Measures the extent to which the material guides the patient, after reviewing it, towards concrete, clear and applicable steps. The main elements examined are: Concrete actions — the material states clearly exactly what the patient should do about their own health or care (for example, which symptom warrants calling whom, or how a medication is to be used); User action — the material offers the patient small, manageable steps; Defining the process — the material clearly describes the path the patient is to follow in actions they can carry out themselves (self-care or follow-up).

## PEMAT Scoring System: Each item is scored as "1 (Yes / criterion met)" or "0 (No / criterion not met)". Items that do not appear in the material, or that are not applicable to it, are marked "N/A" (Not Applicable) and are left out of the assessment. The domain score is then obtained by summing the items scored 1, dividing that sum by the total number of applicable items assessed, and multiplying by 100, giving the Understandability (%) and Actionability (%) scores

## Output Format and Rules: When I provide you with transcripts, you will derive clear, objective, and justified scores for each transcript according to the criteria above. At the very end, you will summarize each score type (DISCERN, GQS, PEMAT-U %, and PEMAT-A %) in a clear Markdown Table so that we can neatly compare all results.

The prompt stated the criteria and scoring rules of each instrument, as reproduced above. The DISCERN criteria and the PEMAT domain criteria are given in the summary form in which they appeared in the prompt; the published item wording of DISCERN and the individual items of the PEMAT understandability and actionability domains were not reproduced in the prompt. No item was removed from any instrument in advance. The expert raters scored from the published instrument forms. The constraint on visual elements was worded generally, across instruments, and offered "Not Applicable / N/A" or "No (0)" as the response to an element absent from the transcript; on the DISCERN 1-to-5 scale neither option is available, and every DISCERN total returned by every rater, expert and model, fell within the 16–80 range of the complete instrument.

## **S2.3 Output**

The prompt specified the output format: after evaluating the transcripts, the model was to summarize the four score types (DISCERN, GQS, PEMAT-Understandability %, and PEMAT-Actionability %) in a single Markdown table so that results could be compared directly. Item-level scores were not requested in the output and were not retained. The instrument totals given in that table were transcribed into the study data form by the authors.

## **S2.4 Session procedure**

In each run, the prompt was entered at the start of a new session, and the 90 anonymized transcripts (V001–V090) were then submitted one after another, in the same order, within that session. Each model completed three runs in three separate sessions, with conversation memory cleared before each run. ChatGPT-5.5, Claude Opus 4.8 and Gemini 3.1 Pro were accessed through their public web interfaces with default settings; in those interfaces "clearing conversation memory" means starting a new conversation with no carried-over context and with any stored personalization disabled. MedGemma 27B (google/medgemma-27b-text-it) was run in Google Colab with the same prompt and the default generation settings of the checkpoint. Because this is a notebook rather than a chat interface, the session structure differed: a script implementing the same prompt was written in Colab, and the transcripts were then evaluated one at a time, each in a separate generation call consisting of the prompt followed by that transcript alone, with no conversation history carried from one transcript to the next. The sequential-session structure described above therefore applies to the three web interfaces only. MedGemma repeated this per-transcript procedure in each of the three runs. All model evaluations were performed on 29 May 2026. Run-level results are given in Supplementary Tables S4 and S5.

## **S2.5 Pilot**

Before the main evaluation, the prompt was piloted on the first 10 transcripts (V001–V010) to confirm that each model followed the instructions and returned a total score for every instrument. No changes to the prompt were required. The pilot outputs were not used in any analysis: all 90 transcripts, including V001–V010, were rescored from the beginning in each of the three main runs. The expert scores were not consulted during the pilot, and the prompt was not adjusted to increase agreement with the expert panel.

# **Section S3. Supplementary Tables**

**Supplementary Table S1. Model access, versions and evaluation conditions.**

| **Field** | **ChatGPT-5.5** | **Claude Opus 4.8** | **Gemini 3.1 Pro** | **MedGemma 27B** |
| --- | --- | --- | --- | --- |
| Access method | Web interface (chatgpt.com) | Web interface (claude.ai) | Web interface (gemini.google.com) | Google Colab (Hugging Face) |
| Model / variant | ChatGPT-5.5 | Claude Opus 4.8 | Gemini 3.1 Pro | MedGemma 27B, instruction-tuned (google/medgemma-27b-text-it) |
| Evaluation date | 29 May 2026 | 29 May 2026 | 29 May 2026 | 29 May 2026 |
| Session handling | 3 runs; each run in a new session with conversation memory cleared; prompt given once, then the 90 transcripts submitted sequentially in the same order | As above | As above | 3 runs; prompt and one transcript per generation call; no conversation history carried between transcripts |
| Input per run | Prompt once, then the 90 transcripts in sequence | Same | Same | Colab script implementing the prompt; prompt plus one transcript per generation call, repeated for all 90 transcripts |
| Decoding (temperature, top-p) | Not user-configurable; not recorded | Same | Same | Default settings of the checkpoint; not recorded |
| Input length / truncation | Full transcript; no truncation applied | Same | Same | Same |
| Web search / tools | Instructed not to use; not verified as disabled | Same | Same | None (no retrieval or tools) |
| Memory / personalization | Conversation memory cleared before each run | Same | Same | Not applicable |
| Repeat runs | Three runs; mean of the runs, rounded to the scoring granularity of the instrument | Same | Same | Same |
| Implementation notes | — | — | — | HF checkpoint run in Colab |

All models received the same English-language prompt (Supplementary Section S2) and were asked to report the total score of each instrument for each transcript. Decoding parameters are not exposed by the web interfaces, and default reasoning depth was not controlled.

**Supplementary Table S2. Bias and limits of agreement of each model relative to expert consensus (n = 90).**

| **Instrument** | **Model** | **Mean bias (95% CI)** | **95% limits of agreement** |
| --- | --- | --- | --- |
| DISCERN | ChatGPT-5.5 | −0.90 (−2.10 to 0.29) | −12.28 to 10.49 |
| DISCERN | Claude | −0.93 (−2.51 to 0.59) | −15.60 to 13.74 |
| DISCERN | Gemini | +13.03 (10.19 to 15.79) | −13.39 to 39.44 |
| DISCERN | MedGemma | +10.93 (8.51 to 13.39) | −11.60 to 33.45 |
| GQS | ChatGPT-5.5 | +0.26 (0.14 to 0.37) | −0.82 to 1.34 |
| GQS | Claude | +0.15 (0.01 to 0.28) | −1.14 to 1.44 |
| GQS | Gemini | +0.94 (0.77 to 1.11) | −0.75 to 2.62 |
| GQS | MedGemma | +0.47 (0.31 to 0.63) | −1.01 to 1.95 |
| PEMAT-A | ChatGPT-5.5 | −0.69 (−2.75 to 1.42) | −20.98 to 19.60 |
| PEMAT-A | Claude | −3.12 (−5.89 to −0.25) | −29.67 to 23.43 |
| PEMAT-A | Gemini | +18.36 (14.41 to 22.00) | −17.80 to 54.53 |
| PEMAT-A | MedGemma | +7.40 (4.20 to 10.69) | −24.38 to 39.17 |
| PEMAT-U | ChatGPT-5.5 | −4.61 (−7.15 to −2.08) | −28.39 to 19.18 |
| PEMAT-U | Claude | −5.77 (−10.31 to −1.61) | −48.14 to 36.59 |
| PEMAT-U | Gemini | +9.13 (3.81 to 14.52) | −43.15 to 61.40 |
| PEMAT-U | MedGemma | +7.43 (2.98 to 12.12) | −37.46 to 52.32 |

Bias = model score − expert-consensus score (mean of three oncologists); positive values indicate higher model scores. Limits of agreement = mean bias ± 1.96 SD of the paired differences. Bias confidence intervals are percentile intervals from 2,000 bootstrap resamples of transcripts. Proportional-bias slopes are not reported: regressing the difference between two measurements on their mean yields a non-zero slope whenever the measurements differ in precision, which cannot be excluded here, so such slopes would not be interpretable. PEMAT-A/-U, PEMAT-Actionability/-Understandability.

**Supplementary Table S3. Mean absolute error of each model from expert consensus, by instrument (values underlying Figure 2).**

| **Instrument** | **ChatGPT-5.5** | **Claude** | **Gemini** | **MedGemma** | **Friedman χ²** | **p** |
| --- | --- | --- | --- | --- | --- | --- |
| DISCERN | 4.65 (3.95–5.42) | 5.63 (4.66–6.72) | 16.11 (14.16–18.07) | 13.58 (11.93–15.27) | 115.4 | < 0.001 |
| GQS | 0.46 (0.38–0.54) | 0.50 (0.41–0.59) | 1.05 (0.90–1.20) | 0.67 (0.55–0.80) | 63.4 | < 0.001 |
| PEMAT-A | 7.27 (5.90–8.94) | 10.12 (8.26–12.18) | 21.19 (18.13–24.30) | 14.85 (12.89–16.91) | 54.0 | < 0.001 |
| PEMAT-U | 8.15 (6.14–10.24) | 13.83 (10.41–17.64) | 21.68 (18.02–25.53) | 17.99 (14.63–21.46) | 28.7 | < 0.001 |

*Mean absolute error is expressed in the native units of each instrument; lower values indicate closer agreement with expert consensus. Values in parentheses are 95% percentile bootstrap confidence intervals from resampling of transcripts. Friedman tests were performed across the four models with df = 3; Kendall's W was 0.43 for DISCERN, 0.23 for GQS, 0.20 for PEMAT-Actionability and 0.11 for PEMAT-Understandability, so the effect was substantial for DISCERN and small for PEMAT-Understandability. PEMAT-A/-U, PEMAT-Actionability/-Understandability.*

**Supplementary Table S4. Within-model run-to-run repeatability across three runs (n = 90 transcripts).**

| **Instrument** | **Model** | **ICC(A,1), single run (95% CI)** | **ICC(A,k), mean of 3 runs (95% CI)** | **Within-transcript SD** | **Identical in all 3 runs, %** |
| --- | --- | --- | --- | --- | --- |
| DISCERN | ChatGPT-5.5 | 0.91 (0.88–0.93) | 0.97 (0.96–0.97) | 3.70 | 0.0 |
| DISCERN | Claude | 0.92 (0.89–0.94) | 0.97 (0.96–0.98) | 3.23 | 11.1 |
| DISCERN | Gemini | 0.69 (0.55–0.78) | 0.87 (0.78–0.91) | 6.44 | 1.1 |
| DISCERN | MedGemma | 0.95 (0.93–0.97) | 0.98 (0.97–0.99) | 2.17 | 73.3 |
| GQS | ChatGPT-5.5 | 0.38 (0.26–0.51) | 0.64 (0.51–0.75) | 0.85 | 35.6 |
| GQS | Claude | 0.54 (0.41–0.65) | 0.78 (0.67–0.85) | 0.61 | 36.7 |
| GQS | Gemini | 0.40 (0.25–0.53) | 0.66 (0.51–0.77) | 0.67 | 55.6 |
| GQS | MedGemma | 0.72 (0.60–0.83) | 0.89 (0.82–0.93) | 0.25 | 81.1 |
| PEMAT-A | ChatGPT-5.5 | 0.95 (0.92–0.96) | 0.98 (0.97–0.99) | 3.98 | 16.7 |
| PEMAT-A | Claude | 0.97 (0.95–0.98) | 0.99 (0.98–0.99) | 2.65 | 33.3 |
| PEMAT-A | Gemini | 0.83 (0.71–0.88) | 0.94 (0.88–0.96) | 3.17 | 16.7 |
| PEMAT-A | MedGemma | 0.70 (0.59–0.80) | 0.87 (0.81–0.92) | 4.51 | 63.3 |
| PEMAT-U | ChatGPT-5.5 | 0.99 (0.99–0.99) | 1.00 (1.00–1.00) | 3.31 | 44.4 |
| PEMAT-U | Claude | 0.97 (0.96–0.98) | 0.99 (0.99–0.99) | 4.97 | 27.8 |
| PEMAT-U | Gemini | 0.98 (0.96–0.99) | 0.99 (0.99–1.00) | 3.16 | 15.6 |
| PEMAT-U | MedGemma | 0.98 (0.96–0.99) | 0.99 (0.99–1.00) | 2.72 | 43.3 |

*ICC, two-way intraclass correlation coefficient, absolute agreement; k = 3. Confidence intervals are percentile intervals from 2,000 bootstrap resamples of transcripts. The within-transcript SD is the root mean square of the 90 transcript-level standard deviations across the three runs, expressed in the native units of the instrument. Repeatability is characterized in the main text by the single-measure coefficient ICC(A,1), which corresponds to the agreement expected from one run. PEMAT-A/-U, PEMAT-Actionability/-Understandability.*

**Supplementary Table S5a. Mean absolute error against expert consensus for averaged and single-run model scores.**

| **Instrument** | **Model** | **MAE, mean of 3 runs** | **Run 1** | **Run 2** | **Run 3** | **Single run (average)** | **Between-expert MAE** |
| --- | --- | --- | --- | --- | --- | --- | --- |
| DISCERN | ChatGPT-5.5 | 4.65 | 4.59 | 5.44 | 5.60 | 5.21 | 6.51 |
| DISCERN | Claude | 5.63 | 6.12 | 6.23 | 5.89 | 6.08 | 6.51 |
| DISCERN | Gemini | 16.11 | 15.46 | 16.53 | 17.29 | 16.43 | 6.51 |
| DISCERN | MedGemma | 13.58 | 13.76 | 13.60 | 13.51 | 13.62 | 6.51 |
| GQS | ChatGPT-5.5 | 0.46 | 0.88 | 0.58 | 0.59 | 0.68 | 0.42 |
| GQS | Claude | 0.50 | 0.66 | 0.59 | 0.80 | 0.68 | 0.42 |
| GQS | Gemini | 1.05 | 1.23 | 1.16 | 1.01 | 1.13 | 0.42 |
| GQS | MedGemma | 0.67 | 0.73 | 0.70 | 0.75 | 0.73 | 0.42 |
| PEMAT-A | ChatGPT-5.5 | 7.27 | 7.85 | 7.89 | 7.88 | 7.87 | 5.38 |
| PEMAT-A | Claude | 10.12 | 10.76 | 10.36 | 10.02 | 10.38 | 5.38 |
| PEMAT-A | Gemini | 21.19 | 21.07 | 20.78 | 21.60 | 21.15 | 5.38 |
| PEMAT-A | MedGemma | 14.85 | 15.95 | 14.99 | 14.10 | 15.01 | 5.38 |
| PEMAT-U | ChatGPT-5.5 | 8.15 | 9.64 | 8.80 | 8.86 | 9.10 | 8.11 |
| PEMAT-U | Claude | 13.83 | 15.28 | 14.72 | 14.56 | 14.85 | 8.11 |
| PEMAT-U | Gemini | 21.68 | 21.26 | 21.62 | 22.22 | 21.70 | 8.11 |
| PEMAT-U | MedGemma | 17.99 | 18.12 | 17.98 | 18.18 | 18.09 | 8.11 |

*Between-expert MAE: each oncologist compared with the mean of the other two (leave-one-expert-out), i.e., based on single expert ratings. For Gemini on PEMAT-Actionability the averaged-score MAE (21.19) slightly exceeds the mean of the three run-wise MAEs (21.15). This is a consequence of rounding: the inequality |mean(x) − y| ≤ mean|x − y| holds for the exact mean, whereas the averaged score used here was rounded to whole percentage points. With unrounded run means the value is 21.06 and the inequality holds for every model and instrument. MAE, mean absolute error.*

**Supplementary Table S5b. Lenient composite classification (≥ 3 of 4 criteria) for each single run.**

| **Model** | **Run** | **Sensitivity, %** | **Specificity, %** | **Cohen's κ** | **PABAK** | **FP** | **FN** |
| --- | --- | --- | --- | --- | --- | --- | --- |
| ChatGPT-5.5 | 1 | 94.3 | 87.3 | 0.79 | 0.80 | 7 | 2 |
| ChatGPT-5.5 | 2 | 91.4 | 89.1 | 0.79 | 0.80 | 6 | 3 |
| ChatGPT-5.5 | 3 | 77.1 | 90.9 | 0.69 | 0.71 | 5 | 8 |
| Claude | 1 | 62.9 | 92.7 | 0.58 | 0.62 | 4 | 13 |
| Claude | 2 | 65.7 | 89.1 | 0.57 | 0.60 | 6 | 12 |
| Claude | 3 | 68.6 | 83.6 | 0.53 | 0.56 | 9 | 11 |
| Gemini | 1 | 85.7 | 25.5 | 0.09 | −0.02 | 41 | 5 |
| Gemini | 2 | 91.4 | 30.9 | 0.19 | 0.09 | 38 | 3 |
| Gemini | 3 | 85.7 | 38.2 | 0.21 | 0.13 | 34 | 5 |
| MedGemma | 1 | 94.3 | 45.5 | 0.35 | 0.29 | 30 | 2 |
| MedGemma | 2 | 91.4 | 54.5 | 0.41 | 0.38 | 25 | 3 |
| MedGemma | 3 | 91.4 | 49.1 | 0.36 | 0.31 | 28 | 3 |

*Reference standard: expert-majority lenient classification (35 adequate, 55 inadequate). FP, false positive; FN, false negative; PABAK, prevalence- and bias-adjusted kappa.*

**Supplementary Table S6. Cohen's κ and false-positive counts when a single instrument threshold, or the two PEMAT thresholds jointly, is varied from the prespecified value.**

| **Scenario** | **Definition** | **Expert-adequate, n** | **κ ChatGPT-5.5** | **κ Claude** | **κ Gemini** | **κ MedGemma** | **FP ChatGPT-5.5** | **FP Claude** | **FP Gemini** | **FP MedGemma** |
| --- | --- | --- | --- | --- | --- | --- | --- | --- | --- | --- |
| Primary (prespecified) | Lenient | 35 | 0.82 | 0.62 | 0.11 | 0.39 | 6 | 7 | 43 | 26 |
| Primary (prespecified) | Strict | 24 | 0.86 | 0.73 | 0.61 | 0.71 | 3 | 2 | 14 | 8 |
| DISCERN ≥ 39 | Lenient | 36 | 0.80 | 0.60 | 0.14 | 0.41 | 7 | 8 | 42 | 29 |
| DISCERN ≥ 39 | Strict | 24 | 0.83 | 0.70 | 0.61 | 0.71 | 4 | 3 | 14 | 8 |
| DISCERN ≥ 45 | Lenient | 36 | 0.84 | 0.65 | 0.14 | 0.41 | 5 | 6 | 42 | 29 |
| DISCERN ≥ 45 | Strict | 24 | 0.83 | 0.70 | 0.61 | 0.71 | 4 | 3 | 14 | 8 |
| DISCERN ≥ 57 | Lenient | 32 | 0.81 | 0.57 | 0.11 | 0.39 | 5 | 6 | 44 | 26 |
| DISCERN ≥ 57 | Strict | 21 | 0.68 | 0.53 | 0.53 | 0.64 | 4 | 2 | 17 | 10 |
| DISCERN ≥ 63 | Lenient | 28 | 0.82 | 0.67 | 0.17 | 0.59 | 4 | 3 | 39 | 16 |
| DISCERN ≥ 63 | Strict | 18 | 0.50 | 0.30 | 0.41 | 0.40 | 2 | 3 | 20 | 10 |
| GQS ≥ 3 | Lenient | 38 | 0.84 | 0.63 | 0.12 | 0.32 | 5 | 7 | 42 | 31 |
| GQS ≥ 3 | Strict | 26 | 0.92 | 0.77 | 0.54 | 0.60 | 1 | 2 | 15 | 12 |
| PEMAT (both) ≥ 60% | Lenient | 39 | 0.78 | 0.59 | 0.12 | 0.43 | 7 | 9 | 40 | 25 |
| PEMAT (both) ≥ 60% | Strict | 28 | 0.85 | 0.65 | 0.37 | 0.56 | 4 | 4 | 27 | 15 |
| PEMAT (both) ≥ 80% | Lenient | 27 | 0.71 | 0.56 | 0.04 | 0.31 | 6 | 3 | 50 | 29 |
| PEMAT (both) ≥ 80% | Strict | 13 | 0.65 | 0.48 | 0.41 | 0.55 | 2 | 1 | 18 | 11 |

*Primary thresholds: DISCERN ≥ 51, GQS ≥ 4, PEMAT-Actionability and PEMAT-Understandability ≥ 70%. The expert reference standard was re-derived at each threshold combination. Across the full grid (DISCERN 39/45/51/57/63; GQS 3/4; PEMAT-A 60/70/80%; PEMAT-U 60/70/80%; lenient and strict definitions; 180 combinations), ChatGPT-5.5 had the highest κ in 178 combinations. The two exceptions, in which MedGemma ranked first, were both under the strict definition: DISCERN ≥ 57, GQS ≥ 4, PEMAT-Actionability ≥ 60% and PEMAT-Understandability ≥ 70% (κ 0.70 for MedGemma versus 0.66 for ChatGPT-5.5), and DISCERN ≥ 57, GQS ≥ 4, PEMAT-Actionability ≥ 80% and PEMAT-Understandability ≥ 70% (κ 0.54 versus 0.52). Gemini or MedGemma produced the most false positives in all 180; ChatGPT-5.5 produced fewer false positives than both Gemini and MedGemma in all 180. These counts describe the present dataset and are not inferential; κ is sensitive to prevalence, and the number of expert-adequate transcripts ranges from 13 to 39 across the grid. The complete 180-combination grid is available from the corresponding author on reasonable request.*

**Supplementary Table S7. Rounding sensitivity analysis: primary (rounded) versus unrounded run means.**

| **Outcome** | **Model** | **Rounded mean (primary)** | **Unrounded mean** |
| --- | --- | --- | --- |
| DISCERN MAE | ChatGPT-5.5 | 4.65 | 4.68 |
| DISCERN MAE | Claude | 5.63 | 5.65 |
| DISCERN MAE | Gemini | 16.11 | 16.11 |
| DISCERN MAE | MedGemma | 13.58 | 13.58 |
| GQS MAE | ChatGPT-5.5 | 0.46 | 0.52 |
| GQS MAE | Claude | 0.50 | 0.57 |
| GQS MAE | Gemini | 1.05 | 1.05 |
| GQS MAE | MedGemma | 0.67 | 0.73 |
| PEMAT-A MAE | ChatGPT-5.5 | 7.27 | 7.30 |
| PEMAT-A MAE | Claude | 10.12 | 10.17 |
| PEMAT-A MAE | Gemini | 21.19 | 21.06 |
| PEMAT-A MAE | MedGemma | 14.85 | 14.85 |
| PEMAT-U MAE | ChatGPT-5.5 | 8.15 | 8.29 |
| PEMAT-U MAE | Claude | 13.83 | 13.93 |
| PEMAT-U MAE | Gemini | 21.68 | 21.63 |
| PEMAT-U MAE | MedGemma | 17.99 | 17.98 |
| Lenient composite κ (FP) | ChatGPT-5.5 | 0.82 (6) | 0.79 (6) |
| Lenient composite κ (FP) | Claude | 0.62 (7) | 0.57 (6) |
| Lenient composite κ (FP) | Gemini | 0.11 (43) | 0.13 (42) |
| Lenient composite κ (FP) | MedGemma | 0.39 (26) | 0.41 (25) |
| Strict composite κ (FP) | ChatGPT-5.5 | 0.86 (3) | 0.86 (3) |
| Strict composite κ (FP) | Claude | 0.73 (2) | 0.66 (2) |
| Strict composite κ (FP) | Gemini | 0.61 (14) | 0.67 (11) |
| Strict composite κ (FP) | MedGemma | 0.71 (8) | 0.72 (6) |

*The primary analyses used the mean of the three runs rounded to the scoring granularity of each instrument, as recorded in the study data form; the unrounded column repeats the mean absolute error and composite classification analyses using the exact mean of the three runs. Mean absolute errors differ by at most 0.14 units, and ChatGPT-5.5 remains the closest model to expert consensus on all four instruments under either approach. Under the lenient composite the ranking of all four models is preserved. Under the strict composite ChatGPT-5.5 remains highest, but the ordering of the remaining three models changes, so the strict-composite ordering below ChatGPT-5.5 is not robust to this choice. FP, false positives. MAE, mean absolute error.*

# **Section S4. Supplementary Figures**

Supplementary Figure S1. Study flow. YouTube search (29 May 2026) using ten prespecified English-language queries; the first 50 standard results per query (500 search-result positions before cross-query deduplication) were screened for eligibility. Of the 500 results screened, 410 were excluded as duplicates or ineligible (not in English; not intended for patients or caregivers; unrelated to oncology or immunotherapy; solely promotional; insufficient spoken content or unusable audio); reason-specific counts were not recorded. Ninety videos were retained (V001–V090), and the eligibility of all included videos was verified by a second oncologist. Standardized, anonymized transcripts were then scored independently by three medical oncologists and four large language models, each model in three runs.

Supplementary Figure S2. Distribution of scores by rater for each instrument. Each box represents 90 transcript-level scores. Model scores are the mean of three runs, rounded to the scoring granularity of the instrument. Diamonds indicate means, center lines medians, boxes the interquartile range, and whiskers/points the remaining distribution and outliers. Blue boxes denote the four large language models (LLMs, n = 4) and grey boxes the three expert oncologists (n = 3).

Supplementary Figure S3. Bland–Altman plots comparing each model's DISCERN scores with expert consensus. Solid line = mean bias; dashed lines = 95% limits of agreement; dotted grey line = zero difference. Each point represents one transcript; model scores are the mean of three runs, rounded to the scoring granularity of the instrument, compared with the mean of the three oncologists. A proportional-bias regression line is displayed for visual reference only and is not interpreted: regressing the difference between two measurements on their mean yields a non-zero slope whenever the measurements differ in precision, which cannot be excluded here, so proportional-bias slopes are not reported in Supplementary Table S2.

Supplementary Figure S4. Instrument-level classification performance against the expert-majority adequacy classifications. Sensitivity (A) and specificity (B) are percentages on a shared 0–100 colour scale; Cohen's κ (C) is shown on a separate diverging scale spanning −0.10 to 1.00 so that negative values (e.g., Gemini on PEMAT-Actionability, κ = −0.02) are represented rather than clipped. PEMAT-A, PEMAT-Actionability; PEMAT-U, PEMAT-Understandability.

# **Section S5. Additional Results**

Instrument-level classification showed that ChatGPT-5.5 yielded the highest Cohen's κ across all four instruments and the most balanced sensitivity–specificity profile. Gemini and MedGemma generated particularly high numbers of false positives for DISCERN, GQS and PEMAT-Actionability; on PEMAT-Actionability, Gemini's specificity was 0% and MedGemma's 10%. Composite areas under the ROC curve were 0.97 for ChatGPT-5.5, 0.88 for Claude, 0.85 for MedGemma and 0.78 for Gemini. Because the index score and the reference standard are derived from the same four thresholds, these curves re-express the composite classification of Table 4 rather than providing independent validation, and with only five possible score levels (0–4 criteria met) the areas should be interpreted cautiously.

In exploratory analyses, the expert DISCERN score correlated neither with view count (ρ = 0.07, p = 0.51) nor like count (ρ = 0.18, p = 0.09), and view count was unrelated to GQS (ρ = 0.08, p = 0.43). Because raw counts do not account for video age (median 2,392 days; range 175–4,398), age-adjusted metrics were examined: views per day showed no significant association with expert DISCERN (ρ = 0.16, p = 0.13), whereas the likes-to-views ratio correlated modestly with expert DISCERN (ρ = 0.28, p = 0.009) and expert GQS (ρ = 0.29, p = 0.006). Video duration correlated moderately with expert DISCERN (ρ = 0.59, p < 0.001).
